# In-Hospital Adverse Events of Pheochromocytoma-Induced Takotsubo Syndrome: A Literature Review and Cluster Analysis of 172 Cases

**DOI:** 10.31083/j.rcm2506216

**Published:** 2024-06-14

**Authors:** Mei Xu, Qianglin Guan, Tianmin Liu, Yuxi Huang, Cunxue Pan, Liyun Luo, Wenyi Tang, Junwei Xu, Hsi Huang, Li Xiao, Kan Liu, Jian Chen

**Affiliations:** ^1^Department of Cardiovascular Medicine, The Fifth Affiliated Hospital of Sun Yat-sen University, 519000 Zhuhai, Guangdong, China; ^2^Department of Radiology, The Fifth Affiliated Hospital of Sun Yat-sen University, 519000 Zhuhai, Guangdong, China; ^3^Center for Interventional Medicine, The Fifth Affiliated Hospital of Sun Yat-sen University, 519000 Zhuhai, Guangdong, China; ^4^Division of Cardiology, Heart and Vascular Center, Washington University in St Louis, Barnes-Jewish Hospital, St Louis, MO 63110, USA; ^5^Guangdong Provincial Engineering Research Center of Molecular Imaging, The Fifth Affiliated Hospital of Sun Yat-sen University, 519000 Zhuhai, Guangdong, China

**Keywords:** pheochromocytoma, takotsubo syndrome, cluster analysis, symptoms and signs, chest pain

## Abstract

**Background:**

Pheochromocytoma-induced takotsubo syndrome (Pheo-TTS) significantly increases the risk of adverse events for inpatient. The early identification of risk factors at admission is crucial for effective risk stratification and minimizing complications in Pheo-TTS patients.

**Methods:**

We conducted a systematic review combined with hierarchical cluster and feature importance analysis of demographic, clinical and laboratory data upon admission, alongside in-hospital complication data for Pheo-TTS patients. We analyzed cases published in PubMed and Embase from 2 May 2006 to 27 April 2023.

**Results:**

Among 172 Pheo-TTS patients, cluster analysis identified two distinct groups: a chest pain dominant (CPD) group (n = 86) and a non-chest pain dominant (non-CPD) group (n = 86). The non-CPD group was characterized by a younger age (44.0 ± 15.2 *vs*. 52.4 ± 14.4, *p* < 0.001), a higher prevalence of neurological/psychiatric disorders (53.5% *vs*. 32.6%), and increased presentation of dyspnea (87.2% *vs*. 17.4%), pulmonary rales (59.3% *vs*. 8.1%), and tachycardia (77.9% *vs*. 30.2%). Additionally, they exhibited more atypical takotsubo syndrome (TTS) imaging phenotypes (55.8% *vs*. 36.5%, all *p* < 0.05). The non-CPD group experienced more than a 2-fold increase for in-hospital adverse events compared to the CPD group (70.9% *vs*. 30.2%, *p* < 0.001). After adjusting for confounding factors, the absence of chest pain (odds ratio [OR] = 0.407, 95% confidence interval [CI] 0.169–0.979, *p* = 0.045), the presence of abdominal symptoms (OR = 3.939, 95% CI 1.770–8.766, *p* = 0.001), pulmonary rales (OR = 4.348, 95% CI 1.857–10.179, *p* = 0.001), and atypical TTS imaging phenotype (OR = 3.397, 95% CI 1.534–7.525, *p* = 0.003) remained as independent predictors of in-hospital complications.

**Conclusions:**

Clinical manifestations and imaging features at admission help to predict in-hospital complications for Pheo-TTS patients.

## 1. Introduction

Pheochromocytoma is a catecholamine-producing neuroendocrine tumor arising from chromaffin cells [1]. Recent studies have highlighted increases in Takotsubo syndrome (TTS) triggered by pheochromocytoma (Pheo-TTS), which have even led to updates in the TTS diagnostic criteria [2, 3, 4, 5]. Despite the growing awareness, the precise incidence of Pheo-TTS and its link to severe in-hospital outcomes remains poorly understood, with numerous studies documenting significant adverse events [6, 7, 8]. The relationship between clinical imaging features observed at admission and the subsequent risk of adverse events and outcomes is particularly unclear. This study aims to explore the potential association between clinical predictors at admission and the occurrence of inpatient complications in Pheo-TTS patients, facilitating better risk stratification and potentially mitigating adverse events.

In this study, we utilized cluster analysis to categorize patients with Pheo-TTS, an approach particularly effective for mapping different clinical phenotypes, pheno-mapping, across a wide spectrum of demographic, clinical and imaging data [6, 9]. This method facilitates the development of targeted preventive and therapeutic strategies ultimately aiming to improve outcomes [6, 9]. Specifically, we conducted an unsupervised, data-driven hierarchical cluster analysis on published Pheo-TTS cases, focusing on signs and admission symptoms. Our goal was to pinpoint unique demographic, clinical, and imaging characteristics present at admission that are predictive of subsequent adverse events among Pheo-TTS patients.

## 2. Materials and Methods

### 2.1 Study Populations 

We collected all case reports from PubMed and the Embase database encompassing dates up to April 27, 2023. The search strategy employed for PubMed was: “ ‘Cardiomyopathy, Takotsubo’ OR ‘Tako-Tsubo Syndrome’ OR ‘Syndrome, Tako-Tsubo’ OR ‘Tako Tsubo Syndrome’ OR ‘Tako-Tsubo Syndromes’ OR ‘Left Ventricular Apical Ballooning Syndrome’ OR ‘Broken Heart Syndrome’ OR ‘Takotsubo Syndrome’ OR ‘Transient Apical Ballooning Syndrome’ OR ‘Apical Ballooning Syndrome’ OR ‘Tako-Tsubo Cardiomyopathy’ OR ‘Cardiomyopathy, Tako-Tsubo’ OR ‘Tako Tsubo Cardiomyopathy’ OR ‘Tako-Tsubo Cardiomyopathies’ OR ‘Stress Cardiomyopathy’ OR ‘Cardiomyopathy, Stress’ AND ‘Pheochromocytomas’ OR ‘Pheochromocytoma, Extra-Adrenal’ OR ‘Extra-Adrenal Pheochromocytoma’ OR ‘Extra-Adrenal Pheochromocytomas’ OR ‘Pheochromocytoma, Extra Adrenal’ OR ‘Pheochromocytomas, Extra-Adrenal’ ”. In Embase, it was: “ ‘Ampulla Cardiomyopathy’ OR ‘Apex Ballooning’ OR ‘Apical Ballooning’ OR ‘Apical Ballooning Syndrome’ OR ‘Broken Heart Syndrome’ OR ‘Left Ventricular Apical Ballooning’ OR ‘Left Ventricular Apical Ballooning Syndrome’ OR ‘Left Ventricular Ballooning’ OR ‘Stress Cardio-myopathy’ OR ‘Stress Cardiomyopathy’ OR ‘Stress Induced Cardiomyopathy’ OR ‘Stress-induced Cardio-myopathy’ OR ‘Tako Tsubo Cardiomyopathy’ OR ‘Tako-Tsubo’ OR ‘Tako-Tsubo Syndrome’ OR ‘Takotsubo’ OR ‘Takotsubo Syndrome’ OR ‘Transient Left Ventricular Apical Ballooning’ OR ‘Transient Left Ventricular Apical Ballooning Syndrome’ OR ‘Takotsubo Cardiomyopathy’ AND ‘Catecholamine-producing Neuroendocrine Tumor’ OR ‘Catecholamine-producing Tumour’ OR ‘Catecholamine-secreting Neuroendocrine Tumor’ OR ‘Catecholamine-secreting Tumor’ OR ‘Catecholamine-secreting Tumour’ OR ‘Epinephrine secreting Tumor’ OR ‘Norepinephrine secreting Tumor’ OR ‘Paraganglioma/Pheochromocytoma’ OR ‘PCC/PGL’ OR ‘PGL/PCC’ OR ‘Pheochromocytoma/Paraganglioma’ OR ‘Catecholamine-producing Tumor’ ”. Exclusion criteria included the absence of Pheo-TTS, ambiguous diagnosis, duplication, other language (not English, German or Chinese) or insufficient information.

Articles were initially screened for titles and abstracts, and full-text articles of potentially relevant reports were reviewed. Reference lists of retrieved full-text studies were scanned to identify additional relevant reports. Only case reports or case series with sufficient information on each case were included. Two researchers searched for Pheo-TTS cases, which were reviewed by senior experts before being summarized.

### 2.2 Demographic, Symptomatic, and Auxiliary Examination Data 

Demographic data including age, gender and history of cardiovascular risk factors were incorporated into the study. Data on symptoms and signs were recorded in detail, such as neurological and/or psychiatric disorders (dizziness, headache, unconsciousness, or others such as drowsiness, mental agitation, panic, sensory and motor disorders), dyspnea, chest pain symptoms (defined as chest pain, chest tightness or radiating pain), abdominal symptoms (nausea, vomiting, abdominal pain or diarrhea), sweating, and other symptoms (pallor, palpitations, fever or weakness). Tachycardia was defined as an increased heart rate of more than 100 beats per minute. Auxiliary examination data were collected, including electrocardiogram (ECG) information on ST-segment elevation or depression, and T-wave inversion. In order to establish a definitive diagnosis of pulmonary edema, chest computed tomography (CT) or X-ray examinations were reviewed. Transthoracic echocardiography (TTE) was used to assess regional wall motion abnormalities, which were further categorized into typical apical TTS and its atypical forms (global, midventricular, basal or focal). In-hospital complications including the administration of catecholamines, development of cardiogenic shock, requirement for invasive or non-invasive ventilation, occurrence of cardiopulmonary resuscitation, and death from any cause were recorded [10]. We compared the demographic, clinical and imaging features of these patients with those in the general TTS population from the International Takotsubo Registry (InterTAK Registry) [10].

### 2.3 Cluster Analysis

Hierarchical cluster analysis was performed according to the clustering variables of admission symptoms and signs (neurological and/or psychiatric disorders, dyspnea, chest pain, abdominal symptoms, sweating, pulmonary rales, and tachycardia) (**Supplementary Table 1** and **Supplementary Fig. 1**).

### 2.4 Statistical Analysis 

Continuous variables were presented as mean ± standard deviation or median (interquartile range) and compared using Student’s *t*-test for normally distributed data or Mann-Whitney U test for non-normally distributed data. Categorical variables were presented as numbers (percentages). The Chi-squared test was used for categorical variables with all cell counts ≥5 and Fisher’s exact test for categorical variables with any cell counts <5. Logistic regression analysis was performed to evaluate factors associated with in-hospital complications. All analyses were performed with SPSS version 26.0 (IBM Corp., Armonk, NY, USA). Statistical significance was defined as *p* value < 0.05.

## 3. Results

### 3.1 Overall Study Cohort

**Fig. 1.**
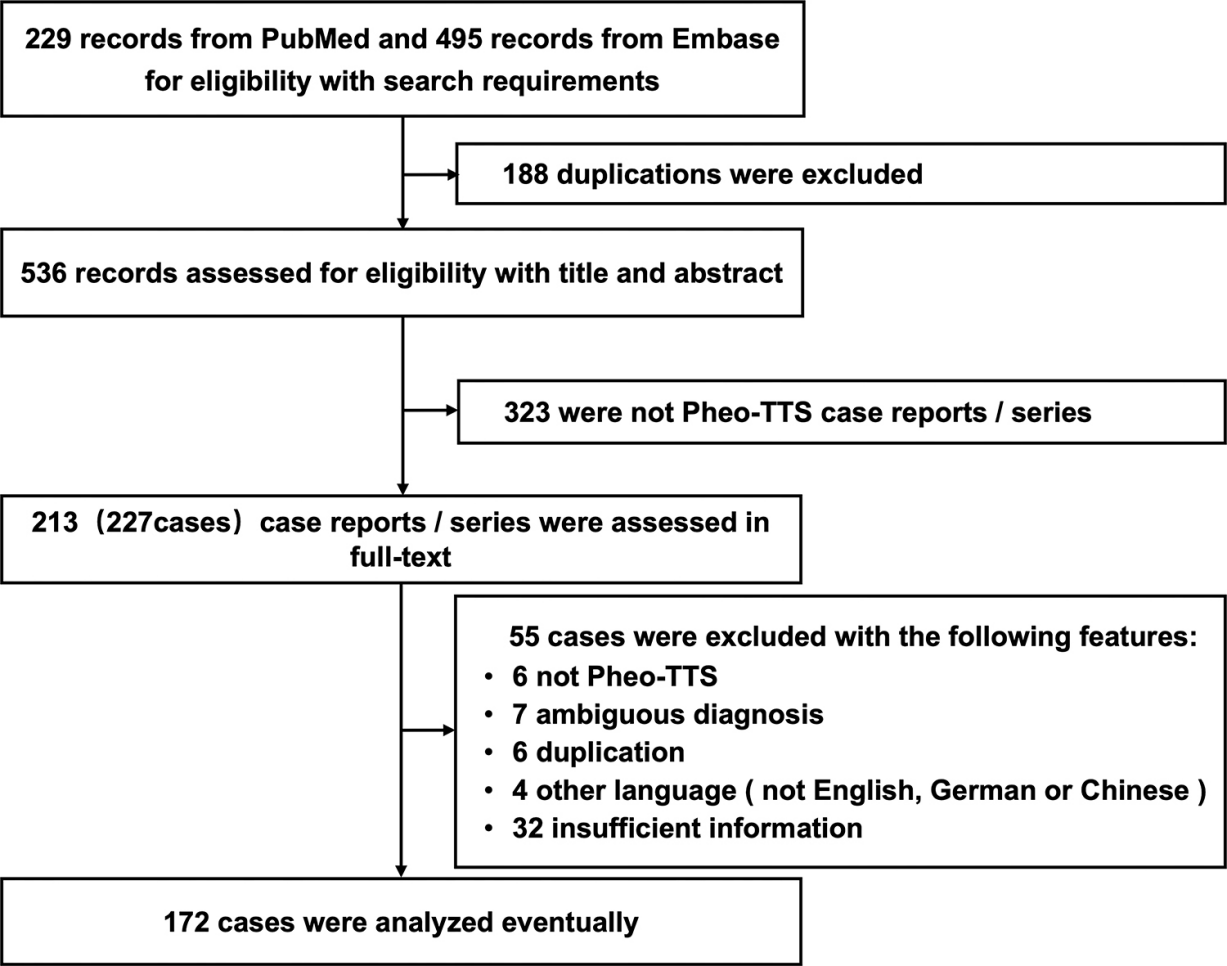
**Selection process of Pheo-TTS cases for study inclusion. **This flowchart illustrates the methodology employed to select cases of pheochromocytoma-induced Pheo-TTS for inclusion in the study. Beginning with an initial screening of 724 articles from PubMed and Embase, the chart details the criteria applied at each step, including eligibility based on diagnostic criteria and completeness of clinical data, culminating in the final selection of 172 patients for analysis. Pheo-TTS, pheochromocytoma-induced takotsubo syndrome.

We screened 229 articles from PubMed and 495 articles from Embase for eligibility based on our search criteria. Of these, 172 patients met the diagnostic criteria for Pheo-TTS and provided complete information on clinical manifestations and diagnostic information during hospitalization (Fig. 1). The cohort predominantly consisted of women (72.1%), with a mean age of 48.2 ± 15.4 years (ranging from 16 to 86 years). The prevalences of hypertension and diabetes within this group were 35.5% and 13.4%, respectively.

The predominant symptom presented by Pheo-TTS patients was chest pain (66.9%), followed by dyspnea (52.3%), abdominal symptoms (47.1%), neurological and/or psychiatric disorders (43.0%), and sweating (30.2%). Less frequent symptoms included pallor, palpitations, fever and weakness. On admission, 85 patients (61.2%) exhibited hypertension, whereas 17 patients (12.1%) presented with hypotension. Tachycardia was observed in more than half of the patients (54.1%), while pulmonary rales were present in nearly one-third (33.7%). ECG analysis revealed ST-segment elevation in 42.6% of cases, ST-segment depression in 29.1%, and T-wave inversion in 17.6%. TTE identified typical (53.8%) and atypical (46.2%) imaging phenotypes. Pulmonary edema was diagnosed by chest CT or X-ray in 57 patients (33.3%).

In-hospital complications occurred in 87 patients (50.6%), with 45.9% requiring invasive or non-invasive ventilation, 37.8% developing cardiogenic shock, 35.5% requiring administration of catecholamines, 16.9% requiring cardiopulmonary resuscitation, and 6.4% resulting in mortality.

Compared to the general TTS population in the InterTAK database, patients with Pheo-TTS presented with distinct demographic and clinical characteristics. Specifically, Pheo-TTS patients were younger (48.2 ± 15.4 *vs*. 66.4 ± 13.1, *p* < 0.001) and more likely to be male (27.9% *vs*. 10.2%, *p* < 0.001). They were also less likely to report chest pain (33.1% *vs*. 24.1% *p* = 0.01), and exhibited a greater prevalence of atypical TTS imaging phenotypes (46.2% *vs*. 18.3%, *p* < 0.001). Moreover, Pheo-TTS patients experienced a more than a 2.3-fold higher incidence of in-hospital complications (50.6% *vs*. 21.8%, *p* < 0.001) (**Supplementary Fig. 2A–E**) [10].

### 3.2 Cluster Analysis

The cluster analysis, based on clinical manifestations at admission, identified in two distinct classifications: a chest pain dominant group (CPD group, n = 86, 50.0%) and a non-chest pain dominant group (non-CPD group, n = 86, 50.0%). The non-CPD group was characterized by younger patients (44.0 ± 15.2 *vs*. 52.4 ± 14.4, *p* < 0.001), and a lower prevalence of hypertension (27.9% *vs*. 43.0%, *p* = 0.038) and diabetes (8.1% *vs*. 18.6%, *p* = 0.044). This group also exhibited a higher incidence of dyspnea (87.2% *vs*. 17.4%, *p* < 0.001), neurological and/or psychiatric disorders (53.5% *vs*. 32.6%, *p* = 0.006), tachycardia (77.9% *vs*. 30.2%, *p* < 0.001) and pulmonary rales (59.3% *vs*. 8.1%, *p* < 0.001) at admission. Furthermore, the non-CPD group had a greater occurrence of pulmonary edema and a lower frequency of ST-segment elevation on ECG (32.9% *vs*. 52.0%, *p* = 0.019), but presented with atypical TTS imaging phenotypes more often (58.0% *vs*. 37.3%, *p* = 0.031). Notably, adverse in-hospital complications were significantly more common in the non-CPD group compared to the CPD group (70.9% *vs*. 30.2%, *p* < 0.001) (Table 1).

**Table 1.** ** Clinical characteristics of Pheo-TTS**.

Characteristics			All	Chest pain dominant group	Non-chest pain dominant group	*p*-value
Patients (n, %)			172 (100.0)	86 (50.0)	86 (50.0)	-
Age (years)			48.2 ± 15.4	52.398 ± 14.4	44.0 ± 15.2	<0.001
Age ≤50 years (n, %)			90/171 (52.6)	35/85 (41.2)	55/86 (64.0)	0.003
Female (n, %)			124/172 (72.1)	60/86 (69.8)	64/86 (74.4)	0.497
Cardiovascular risk factors/Medical history						
	Smoking		20/172 (11.6)	12/86 (14)	8/86 (9.3)	0.341
	Hypertension (n, %)		61/172 (35.5)	37/86 (43.0)	24/86 (27.9)	0.038
	Diabetes (n, %)		23/172 (13.4)	16/86 (18.6)	7/86 (8.1)	0.044
	Hyperlipidemia (n, %)		16/172 (9.3)	9/86 (10.5)	7/86 (8.1)	0.600
	Coronary artery disease (n, %)		4/172 (2.3)	3/86 (3.5)	1/86 (1.2)	0.621
Symptoms on admission						
	Chest pain (n, %)		115/172 (66.9)	66/86 (76.7)	49/86 (57.0)	0.006
	Dyspnea (n, %)		90/172 (52.3)	15/86 (17.4)	75/86 (87.2)	<0.001
	Neurological and/or psychiatric disorders (n, %)		74/172 (43.0)	28/86 (32.6)	46/86 (53.5)	0.006
		Dizzy (n, %)	16/172 (9.3)	8/86 (9.3)	8/86 (9.3)	1.000
		Headache (n, %)	46/172 (26.7)	16/86 (18.6)	30/86 (34.9)	0.016
		Unconsciousness (n, %)	12/172 (7.0)	3/86 (3.5)	9/86 (10.5)	0.132
		Others (n, %)	16/172 (9.3)	5/86 (5.8)	11/86 (12.8)	0.115
	Abdominal symptoms (n, %)		81/172 (47.1)	36/86 (41.9)	45/86 (52.3)	0.169
		Nausea (n, %)	46/172 (26.7)	21/86 (24.4)	25/86 (29.1)	0.491
		Vomiting (n, %)	60/172 (34.9)	26/86 (30.2)	34/86 (39.5)	0.201
		Abdominal pain (n, %)	33/172 (19.2)	15/86 (17.4)	18/86 (20.9)	0.561
		Diarrhea (n, %)	5/172 (2.9)	3/86 (3.5)	2/86 (2.3)	1.000
	Sweating (n, %)		52/172 (30.2)	23/86 (26.7)	29/86 (33.7)	0.319
	Others (n, %)		68/172 (39.5)	28/86 (32.6)	40/86 (46.5)	0.061
Signs on admission						
	Hypertension (n, %)		85/139 (61.2)	42/68 (61.8)	43/71 (60.6)	0.885
	Hypotension (n, %)		17/139 (12.1)	8/68 (11.8)	9/71 (12.7)	0.870
	Hypertension/hypotension (n, %)		100/139 (71.9)	48/68 (70.6)	52/71 (73.2)	0.728
	Tachycardia (n, %)		93/172 (54.1)	26/86 (30.2)	67/86 (77.9)	<0.001
	Pulmonary rales (n, %)		58/172 (33.7)	7/86 (8.1)	51/86 (59.3)	<0.001
ECG						
	ST-segment elevation (n, %)		63/148 (42.6)	39/86 (52.0)	24/86 (32.9)	0.019
	ST-segment depression (n, %)		43/148 (29.1)	18/86 (24.0)	25/86 (34.2)	0.170
	T-wave inversion (n, %)		26/148 (17.6)	12/86 (16.0)	14/86 (19.2)	0.611
Echocardiography						
	Atypical Takotsubo type (n, %)		79/171 (46.2)	31/85 (36.5)	48/86 (55.8)	0.011
	LVEF <30% (n, %)		61/124 (49.2)	24/58 (41.4)	37/66 (56.1)	0.103
Chest CT or X-ray						
	Pulmonary edema (n, %)		57/171 (33.3)	9/85 (10.6)	48/86 (55.8)	<0.001
In-hospital complications (n, %)			87/172 (50.6)	26/86 (30.2)	61/86 (70.9)	<0.001
	Catecholamine use (n, %)		61/172 (35.5)	17/86 (19.8)	44/86 (51.2)	<0.001
	Cardiogenic shock (n, %)		65/172 (37.8)	19/86 (22.1)	46/86 (53.5)	<0.001
	Invasive or noninvasive ventilation (n, %)		79/172 (45.9)	21/86 (24.4)	58/86 (67.4)	<0.001
	Cardiopulmonary resuscitation		29/172 (16.9)	9/86 (10.5)	20/86 (23.3)	0.025
	Death (n, %)		11/172 (6.4)	4/86 (4.7)	7/86 (8.1)	0.535

Pheo-TTS, pheochromocytoma-induced takotsubo syndrome; ECG, electrocardiogram; LVEF, left ventricular ejection fraction; CT, computed tomography.

### 3.3 Prognostication/Outcome Correlates

**Fig. 2.**
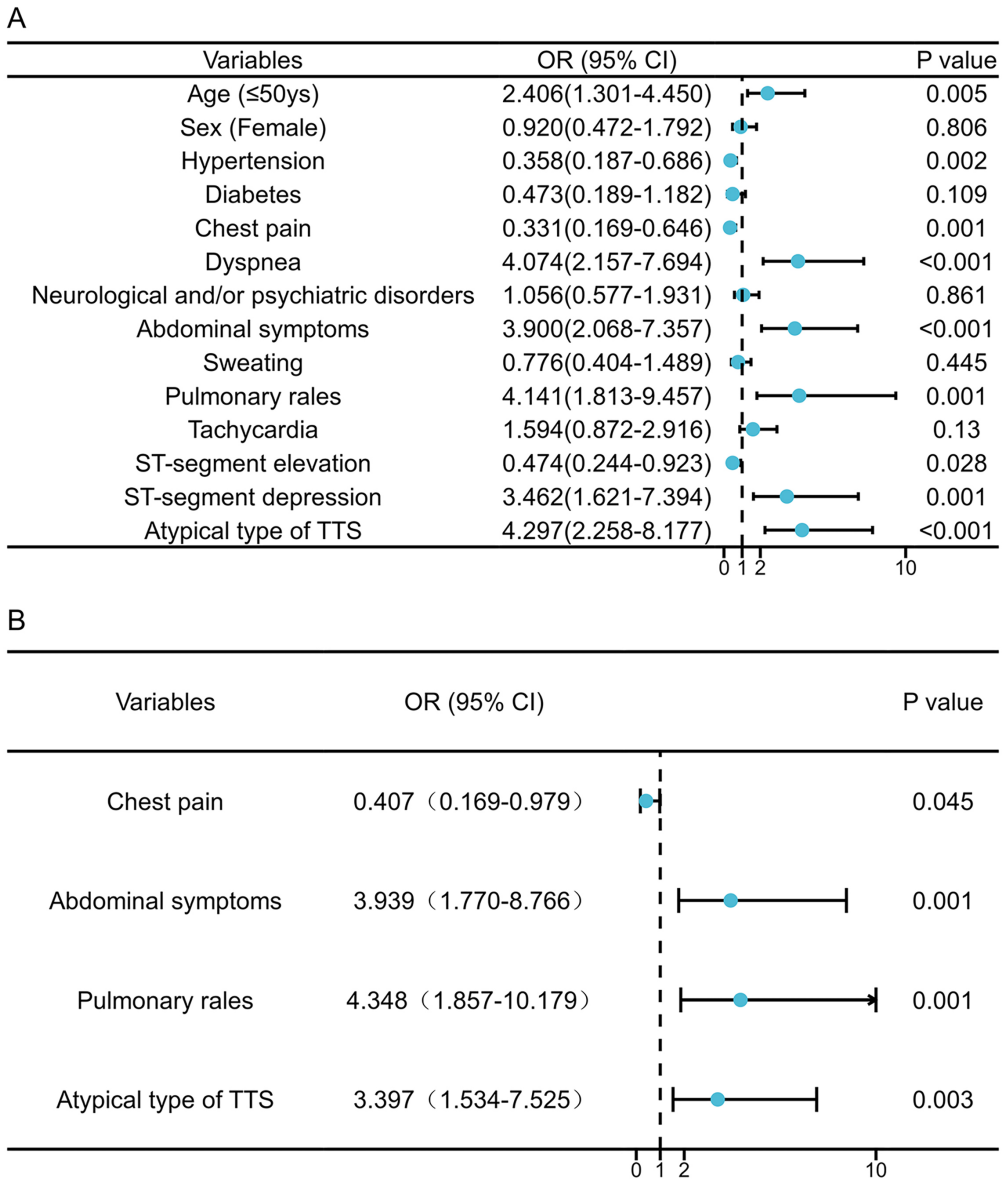
**Factors associated with in-hospital complications of pheochromocytoma-Induced takotsubo syndrome patients.** Univariate (A) and multivariable (B) Cox regression analysis. OR, odds ratio; CI, confidence interval; TTS, takotsubo syndrome.

Univariate logistic regression analysis identified several factors associated with an increased risk of in-hospital complications: a younger age (≤50 years), experiencing dyspnea, abdominal symptoms, pulmonary rales, ST-segment depression, and atypical TTS imaging phenotypes (all *p* < 0.05). Conversely, a history of hypertension, the presence of chest pain, and ST-segment elevation were correlated to a lower risk of in-hospital complications (*p* < 0.05) (Fig. 2A). After adjusting for confounders, a multivariate analysis showed that the absence of chest pain (odds ratio [OR] = 0.407, 95% confidence interval [CI] 0.169–0.979, *p* = 0.045), the presence of abdominal symptoms (OR = 3.939, 95% CI 1.770–8.766, *p* = 0.001), pulmonary rales (OR = 4.348, 95% CI 1.857–10.179, *p* = 0.001) and atypical TTS imaging phenotype (OR = 3.397, 95% CI 1.534–7.525, *p* = 0.003) remained independent predictors of in-hospital complications (Fig. 2B).

## 4. Discussion

The present study highlights several key insights into Pheo-TTS: (1) Compared with the general TTS population, Pheo-TTS patients were significantly younger, more likely to be male, and exhibited a higher rate of in-hospital complications. (2) Through cluster analysis, we identified a distinct subgroup within Pheo-TTS patients who lacked chest pain at presentation, and experienced a significantly higher number of in-hospital complications. (3) Key predictors of increased in-hospital adverse events include the absence of chest pain, the presence of pulmonary and abdominal symptoms, and an atypical TTS imaging phenotype on admission.

Although the exact pathogenesis of TTS is not fully understood, the most widely accepted mechanism is direct myocardial damage due to excessive catecholamine release, especially in specific neuroendocrine and autonomic disorders such as Pheo-TTS and neurological stress cardiovascular disease [6, 11, 12, 13, 14]. Initially, these conditions were exclusion criteria for TTS diagnosis [6, 11, 12, 13, 14]. Evidence suggests that the catecholamine storm associated with Pheo-TTS leads to poorer in-hospital outcomes when compared to cases of pheochromocytoma, TTS, or Pheo alone [8]. The general TTS population exhibits an in-hospital complication rate of approximately 20% [10, 15], while 7–18% of pheochromocytoma patients may experience crises requiring emergency in-hospital management [16].

In our study, more than half of the Pheo-TTS patients developed in-hospital complications. This high incidence can be explained by the severity of pheochromocytoma [8, 17] and the demographic profile of Pheo-TTS patients, who are generally younger [18] and more often male [19]. This aligns with findings from by Y-Hassan S [2] who analyzed 80 published Pheo-TTS cases showing similar results. However, this contrasts with neurological stress cardiovascular diseases, which are influenced by cerebral damage and are affected by older age, being female, and other factors [13, 20]. Identifying correlates of inpatient outcomes is crucial for risk stratification and tailored management strategies.

Patients experiencing Pheo-TTS exhibit a broad range of clinical presentations, blending features of both pheochromocytoma and TTS, often resulting in nonspecific symptoms [1]. This nonspecificity poses a challenge in pinpointing the underlying pathophysiology and in formulating targeted prevention and therapeutic strategies [21, 22]. Our cluster analysis revealed two primary presentation patterns among Pheo-TTS patients: one group predominantly experiencing chest pain, with the second showing symptoms of dyspnea, tachycardia, and pulmonary rales. Similar findings have been observed in other studies, such as the German-Italian-Spanish Takotsubo (GEIST) registry, indicating that TTS patients presenting with non-chest pain symptoms upon admission are more likely to experience in-hospital complications [18, 23, 24]. In addition, the presence of neurological and/or psychiatric disorders [23] and tachycardia [19] have also been associated with adverse TTS outcomes.

Our study revealed that pulmonary and abdominal symptoms and signs were independent predictors of adverse in-hospital outcomes in Pheo-TTS patients. Specifically, pulmonary rales in Pheo-TTS were associated with pulmonary edema (likely due to poor ventricular function), mitral regurgitation, and/or left ventricular outflow tract obstruction—conditions known to worsen in-hospital outcomes [25, 26]. Whether the increased need for mechanical ventilation in Pheo-TTS patients is attributed to the direct effects of catecholamines on the lung or pulmonary vasculature remains a critical area for future research [27]. Furthermore, abdominal symptoms have been linked to increased mortality during a pheochromocytoma crises, potentially due to more extensive systemic organ damage [16] and elevated catecholamines [28]. Notably, the non-CPD group exhibited a higher prevalence of atypical TTS imaging phenotypes, correlating with an increased for in-hospital complications as reported in previous studies [17, 23]. These findings underscore the importance of non-cardiac manifestations upon admission for predicting in-hospital outcomes for Pheo-TTS, offering valuable insights for improving clinical management and prognosis.

## 5. Study limitation

The main limitation of our study is its retrospective nature. The data were collected and analyzed from published case reports and series, which may introduce an element of selection bias. Since some cases with incomplete data had to be excluded, several potentially important parameters such as cardiac biomarkers, blood and urine catecholamine levels, left ventricular ejection fraction, and medication and device treatment strategies were not included in the final cluster analysis. Furthermore, the need for a larger and more diverse dataset—encompassing diverse ethnicities, gender and age are needed to develop prediction models with broader applicability and to facilitate external validation.

## 6. Conclusions

Clinical and imaging characteristics observed upon admission can serve as valuable predictors of in-hospital complications in Pheo-TTS patients. Notably, the absence of chest pain, alongside the presence of pulmonary rales, abdominal symptoms, and an atypical TTS imaging phenotype, are associated with an increased risk of in-hospital adverse events.

## Data Availability

The datasets used and/or analyzed during the current study are available from the corresponding author on reasonable request.

## Abbreviations

CPD group, chest pain dominant group; CT, computed tomography; ECG, electrocardiogram; non-CPD group, non-chest pain dominant group; Pheo-TTS, pheochromocytoma-induced takotsubo syndrome; TTE, transthoracic echocardiography; TTS, takotsubo syndrome.

## Supplementary Material

Supplementary material associated with this article can be found, in the online version, at https://doi.org/10.31083/j.rcm2506216.
