# Supplementary material for: In-Hospital Adverse Events of Pheochromocytoma-Induced Takotsubo Syndrome: A Literature Review and Cluster Analysis of 172 Cases
Source: Rev Cardiovasc Med. 2024 Jun 14;25(6):216. doi: 10.31083/j.rcm2506216 (PMC11270063; doi:10.31083/j.rcm2506216)
Supplement: Supplementary file 1 [file 2153-8174-25-6-216-s1.zip › Supplementary Material-1.docx]

**Supplementary Tab. 1 Admission symptoms and signs of pheochromocytoma-induced Takotsubo syndrome.**

| The lead author of the papers | Publication date | Age（years） | Sex （0=Male，1=Female） | Neurologic and/or psychiatric disorders | Dyspnea | Chest pain | Abdominal Symptoms | Sweating | Other atypical symptoms | Tachycardia | Pulmonary  rales |
| --- | --- | --- | --- | --- | --- | --- | --- | --- | --- | --- | --- |
| Afana, M. | 2019 | 66 | 1 | + | - | + | + | - | - | + | - |
| Gervais, M. K. | 2015 | 26 | 0 | - | - | + | + | - | - | - | - |
| Chen, M. | 2020 | 70 | 0 | - | + | + | + | + | + | + | + |
| Faria, B. M. M. | 2020 | 26 | 1 | + | + | + | - | - | + | + | + |
| Chiang, Y. L. | 2016 | 70 | 1 | + | + | + | + | + | + | + | - |
| Zilio, F. | 2021 | 41 | 0 | - | + | - | + | - | - | + | + |
| Marino, G. | 2021 | 69 | 0 | - | + | + | - | + | - | + | - |
| Maffe, S. | 2021 | 53 | 0 | - | + | + | - | - | - | + | + |
| Spapen, J. | 2020 | 43 | 1 | + | + | + | + | - | - | - | + |
| Sakul, N. F. N. | 2020 | 23 | 1 | + | + | + | + | - | + | + | + |
| Su, Z. | 2019 | 31 | 1 | + | + | - | + | + | + | + | - |
| Kiamanesh, O. | 2019 | 45 | 1 | + | + | + | - | - | + | + | + |
| Tafreshi, S | 2018 | 26 | 1 | + | + | + | + | - | + | - | + |
| Demea, A. D. | 2018 | 52 | 1 | + | + | + | - | + | - | - | + |
| Chow, L. T. C. | 2018 | 43 | 1 | + | + | + | - | - | + | + | + |
| Gravina, M. | 2017 | 23 | 0 | - | + | + | + | + | - | - | - |
| Contadini, D. | 2017 | 42 | 1 | + | + | + | - | - | - | + | - |
| Tagawa, M. | 2015 | 33 | 1 | + | + | + | + | - | + | - | - |
| Y. Hassan S | 2015 | 68 | 1 | + | - | + | + | + | + | - | - |
| Flam, B. | 2015 | 46 | 1 | + | + | + | + | - | + | + | + |
| Brugts, J. J. | 2015 | 75 | 1 | + | - | + | + | + | - | - | - |
| Kaese, S. | 2013 | 43 | 0 | - | - | - | - | - | + | - | - |
| Iio, K. | 2013 | 29 | 1 | + | + | + | - | - | + | - | + |
| Subramanyam, S. | 2012 | 60 | 0 | - | - | + | - | - | + | - | - |
| Zielen, P. | 2010 | 53 | 0 | - | + | + | + | + | + | - | - |
| Marcovitz, P. A. | 2010 | 43 | 1 | + | - | + | - | - | + | + | - |
| Kimura, S. | 2010 | 36 | 1 | + | + | - | - | - | - | - | - |
| Stephen Kim, MD | 2010 | 30 | 0 | - | + | - | + | - | + | + | + |
| Gujja, K. R. | 2010 | 46 | 1 | + | + | + | - | + | + | - | - |
| Von Bergen, N. H. | 2009 | 17 | 0 | - | + | - | + | - | + | - | + |
| Rossi, A. P. | 2009 | 81 | 1 | + | - | + | + | - | + | - | - |
| Jindal, V. | 2009 | 55 | 0 | - | + | + | - | - | + | - | - |
| Zegdi, R. | 2008 | 51 | 1 | + | + | - | - | - | - | - | + |
| R. Pfister | 2008 | 59 | 1 | + | + | + | + | - | - | - | - |
| Di Valentino, M. | 2008 | 52 | 1 | + | + | + | - | - | - | + | - |
| Takizawa, M. | 2007 | 32 | 0 | - | - | + | - | - | - | + | - |
| Sanchez-Recalde, A. | 2006 | 41 | 1 | + | + | - | + | + | - | + | - |
| Dai, Y. L. | 2020 | 28 | 0 | - | + | + | + | - | + | + | + |
| Kissami, I. | 2021 | 49 | 0 | - | + | + | + | - | - | - | + |
| Jiang, X. | 2021 | 63 | 1 | + | - | + | - | + | - | - | + |
| Dominedo, C. | 2021 | 28 | 1 | + | + | - | + | + | + | + | + |
| Platzer, P. | 2020 | 69 | 1 | + | + | + | - | - | - | - | + |
| Nam, M. C. Y. | 2020 | 70 | 1 | + | - | + | - | - | - | - | - |
| Kayode, O. | 2020 | 72 | 1 | + | - | + | - | - | - | - | - |
| Falcetta, P. | 2020 | 69 | 1 | + | + | + | - | + | + | - | - |
| Touma, T | 2019 | 33 | 1 | + | + | + | - | - | - | + | + |
| Garla, V. V. | 2019 | 55 | 1 | + | + | - | - | - | - | - | - |
| Diaz, B. | 2019 | 50 | 1 | + | - | + | - | - | - | - | - |
| Takeshita, Y. | 2018 | 45 | 0 | - | - | - | + | - | - | - | + |
| Loscalzo, J. | 2018 | 55 | 1 | + | + | + | + | - | + | + | + |
| Hernandez-Montoliu, L. | 2018 | 70 | 1 | + | + | + | - | + | + | - | + |
| Butt, K. | 2018 | 42 | 1 | + | + | - | - | - | + | - | - |
| Petersen, M. H. | 2016 | 62 | 0 | - | - | + | - | - | - | - | - |
| Petersen, M. H. | 2016 | 57 | 1 | + | - | + | - | - | + | + | - |
| Hernandez Ramirez, J. M. | 2016 | 52 | 1 | + | - | - | + | - | - | - | - |
| Sharkey, S. W. | 2015 | 16 | 0 | - | - | + | - | - | - | + | - |
| Sakamoto, K. | 2015 | 64 | 1 | + | - | + | - | - | - | - | - |
| Assefa D, | 2015 | 42 | 1 | + | + | - | - | - | + | + | + |
| Zhu, D. | 2014 | 67 | 1 | + | + | + | + | - | + | - | - |
| Hausen, S. | 2014 | 75 | 0 | - | - | - | - | + | + | + | - |
| Battimelli, A. | 2014 | 63 | 1 | + | + | + | - | - | - | + | + |
| Law, C. | 2014 | 23 | 1 | + | + | + | + | + | - | + | + |
| Demirçelik, M. B. | 2013 | 64 | 0 | - | - | + | - | - | + | + | - |
| Santoro, F. | 2012 | 17 | 1 | + | - | - | + | - | - | + | - |
| Ueda, H. | 2011 | 86 | 0 | - | - | - | + | - | - | - | - |
| Park, J. H. | 2011 | 32 | 0 | - | - | + | - | - | + | + | - |
| Park, J. H. | 2011 | 41 | 1 | + | + | + | - | - | - | + | + |
| Park, J. H. | 2011 | 49 | 0 | - | + | - | - | - | - | + | + |
| Tanriver, Y. | 2010 | 57 | 0 | - | + | + | + | + | - | + | + |
| Di Palma, G. | 2010 | 29 | 1 | + | + | - | + | - | + | + | + |
| Mrdovic, I. | 2008 | 53 | 1 | + | - | + | - | - | - | - | - |
| Dimski, T. | 2008 | 71 | 1 | + | + | - | - | - | - | + | + |
| Kim, H. S. | 2007 | 47 | 0 | - | + | - | - | - | + | - | + |
| Spes, C. | 2006 | 56 | 1 | + | - | + | - | - | + | - | - |
| Rostoff, P. | 2020 | 61 | 0 | - | + | + | - | - | - | + | - |
| Casey, R. T. | 2017 | 37 | 1 | + | - | - | + | + | + | + | - |
| Kobayashi, Y. | 2014 |  | 1 | + | - | + | - | - | - | - | - |
| Schiano, P. | 2007 | 58 | 1 | + | - | + | - | - | - | - | - |
| Wang, W. | 2017 | 68 | 1 | + | - | + | + | + | + | + | + |
| de Souza, F. | 2008 | 31 | 1 | + | + | + | - | - | + | + | + |
| Kumar, S | 2010 | 57 | 1 | + | - | + | + | - | + | + | - |
| Chia, P. L. | 2011 | 69 | 1 | + | + | + | + | + | - | - | - |
| Naderi, N. | 2012 | 31 | 1 | + | + | - | - | + | + | + | - |
| Cardillo, M. T. | 2013 | 28 | 0 | - | - | - | + | + | - | + | - |
| Li, Z. K. | 2019 | 43 | 1 | + | + | + | - | + | + | + | + |
| Lau, E. S. | 2021 | 61 | 0 | - | - | - | - | + | - | - | - |
| Cesaretti, M | 2010 | 74 | 0 | - | + | + | + | + | - | - | - |
| Caballero, A. M. | 2019 | 21 | 1 | + | + | + | - | + | - | + | + |
| Diaz-Roldan J, | 2021 | 57 | 1 | + | + | - | - | - | + | + | - |
| Park, J. Y. | 2012 | 28 | 1 | + | - | + | - | - | - | + | - |
| Awadji, F. B. | 2023 | 61 | 0 | - | - | + | - | - | + | + | + |
| Yang, L. | 2022 | 27 | 1 | + | + | + | + | + | - | + | - |
| Wang, Z. H. | 2022 | 64 | 1 | + | - | + | - | + | - | - | - |
| Turner, L. M. | 2022 | 70 | 1 | + | + | + | + | + | + | - | - |
| Polito, M. V. | 2022 | 61 | 1 | + | - | - | + | - | - | - | + |
| Lyu, T. | 2022 | 54 | 1 | + | + | + | - | + | + | + | - |
| Jenča, D. | 2022 | 37 | 1 | + | + | - | - | - | + | - | - |
| Zhou, F. F. | 2022 | 69 | 0 | - | - | + | + | + | - | - | - |
| Yuan, S. | 2021 | 31 | 0 | - | - | - | + | - | - | + | + |
| Itagane, M. | 2021 | 34 | 1 | + | + | - | + | + | - | + | + |
| Bekelaar, T. | 2021 | 49 | 0 | - | + | - | + | - | + | + | - |
| Sahar, T. | 2021 | 63 | 0 | - | - | + | + | - | - | - | - |
| Shahim, B. | 2019 | 53 | 1 | + | + | + | + | - | + | + | - |
| Wong, T. S. | 2018 | 46 | 1 | + | - | - | + | - | - | - | + |
| Yang, T. H. | 2015 | 54 | 1 | + | - | + | - | - | + | + | - |
| Vagner, H. | 2015 | 55 | 1 | + | - | + | - | - | + | + | + |
| Mikail, N. | 2013 | 52 | 0 | - | - | + | + | + | - | - | - |
| Celebi, H. | 2012 | 66 | 1 | + | - | + | - | - | - | - | - |
| Sheinberg, R. | 2012 | 45 | 1 | + | - | - | + | - | + | - | + |
| Ng, D. Z. | 2023 | 51 | 1 | + | + | - | + | - | - | - | + |
| Kokkapuni, N. | 2022 | 39 | 1 | + | - | + | - | + | - | - | - |
| Trongtorsak, A. | 2021 | 50 | 0 | - | + | + | + | - | + | - | + |
| Schmidt, K. H. | 2017 | 25 | 1 | + | - | + | + | - | + | + | - |
| Leitch, J. K. | 2020 | 24 | 1 | + | + | + | - | + | - | + | + |
| Y-Hassan, S. | 2020 | 61 | 1 | + | - | + | + | - | - | - | - |
| Verrijcken, A. | 2011 | 45 | 1 | + | - | - | + | + | - | + | - |
| Schimpf, K. | 2008 | 38 | 1 | + | + | - | + | + | - | + | - |
| Polito, M. V. | 2015 | 24 | 0 | - | + | - | + | + | + | - | - |
| Odierna, I. | 2022 | 48 | 1 | + | - | - | + | - | - | + | - |
| Nomoto, Y. | 2017 | 61 | 0 | - | + | - | + | - | + | + | + |
| Li, X. | 2017 | 46 | 1 | + | - | + | + | - | - | + | + |
| Lassnig, E. | 2009 | 71 | 1 | + | + | - | + | - | - | + | - |
| Kontrimaviciute, E. | 2020 | 39 | 1 | + | + | - | + | + | + | - | + |
| Gingles, C. | 2010 | 45 | 1 | + | - | + | + | - | - | + | - |
| Frey, S. M. | 2023 | 41 | 1 | + | - | + | - | - | + | + | - |
| Dörschner, M. | 2010 | 31 | 1 | + | - | + | - | + | + | - | - |
| Cho, S. K. | 2014 | 45 | 0 | - | - | + | - | - | - | - | - |
| Bhasin, D. | 2021 | 25 | 1 | + | - | + | - | - | - | + | - |
| Barnsteiner, S. | 2019 | 52 | 1 | + | - | + | - | - | - | - | - |
| Aziz, T. A. | 2018 | 59 | 1 | + | + | + | + | - | - | - | - |
| Klinzing, S. | 2013 | 46 | 1 | + | - | + | + | - | - | - | - |
| Kochi, R. | 2015 | 78 | 1 | + | + | - | - | + | - | + | - |
| Huang, W. | 2016 | 28 | 1 | + | + | - | + | + | - | + | + |
| Mohamed, W. S. A. | 2017 | 44 | 0 | - | - | + | + | + | - | - | - |
| Shiekh, I. | 2017 | 57 | 0 | - | + | + | + | - | - | + | + |
| Garuba, H. | 2018 | 49 | 0 | - | - | + | - | + | + | + | - |
| Shareef Mansour, S. H. | 2018 | 44 | 1 | + | + | - | + | + | - | + | - |
| La Rosa, G. | 2019 | 32 | 1 | + | + | - | + | - | - | + | - |
| Whitler, C. | 2019 | 49 | 1 | + | - | - | + | - | - | + | - |
| Costello, M. | 2020 | 39 | 1 | + | - | + | - | - | - | + | - |
| Perdikis, S. | 2020 | 34 | 1 | + | + | + | + | - | - | + | - |
| Taylor, A. P. | 2020 | 59 | 1 | + | + | + | - | - | - | + | + |
| Temesgen, N. | 2020 | 57 | 1 | + | - | + | - | - | - | - | - |
| Angius, S. | 2021 | 60 | 1 | + | - | + | - | - | - | - | - |
| Kaminsky, L. | 2021 | 61 | 1 | + | - | + | - | - | - | - | - |
| Kaushik, S. | 2021 | 56 | 1 | + | - | + | + | - | - | - | - |
| Scarfone, S. | 2021 | 29 | 1 | + | + | - | - | - | - | + | - |
| Cordeanu, E. M. | 2022 | 57 | 1 | + | - | + | - | + | - | + | - |
| Kerley, R. | 2022 | 39 | 1 | + | + | + | - | - | - | - | - |
| Konstantinidis, I. | 2022 | 61 | 0 | - | - | - | - | - | + | + | - |
| Yeo, J. | 2022 | 42 | 0 | - | + | - | - | - | - | - | - |
| Ajluni, S. | 2023 | 28 | 1 | + | + | + | + | - | - | + | + |
| Moulder, M. P. D. | 2023 | 40 | 1 | + | - | + | - | - | - | + | - |
| Xu, L. | 2023 | 52 | 0 | - | - | + | - | + | - | + | - |
| Zhang, C. H. | 2021 | 60 | 1 | + | - | + | - | - | - | + | - |
| Hassan, S. | 2022 | 44 | 0 | - | - | - | + | - | - | - | - |
| Griffin, S. | 2020 | 30 | 1 | + | + | + | + | - | + | + | + |
| Flores Vergara, G. M. | 2018 | 43 | 1 | + | - | + | - | - | - | - | - |
| van Zwet, C. J. | 2016 | 27 | 1 | + | + | + | + | - | - | + | + |
| Artusi, N. | 2022 | 42 | 1 | + | + | - | - | - | - | + | + |
| Artusi, N. | 2022 | 66 | 1 | + | - | - | - | - | + | + | - |
| Bettini, L. | 2022 | 63 | 1 | + | - | - | - | + | + | - | - |
| Cai, Q. | 2019 | 42 | 0 | - | + | + | - | - | - | + | - |
| Mantes, L. | 2019 | 26 | 1 | + | - | - | + | - | - | - | - |
| Park, S. | 2022 | 29 | 1 | + | + | + | + | - | - | + | - |
| Ribeiro, V. | 2013 | 56 | 1 | + | - | - | + | + | + | + | - |
| Abdul-Wahab, A. | 2019 | 39 | 1 | + | - | - | - | - | - | - | + |
| Abdelrehim, M. | 2021 | 64 | 1 | + | - | + | - | + | + | - | - |
| Byreddy, D. V. | 2017 | 59 | 1 | + | - | - | - | - | + | - | - |
| Grønvold, T. | 2008 | 51 | 1 | + | - | + | - | - | - | + | - |
| Hamilton, R. | 2018 | 72 | 1 | + | + | + | - | - | - | - | - |
| Pena, M. E. | 2013 | 32 | 0 | - | + | + | + | + | + | + | - |


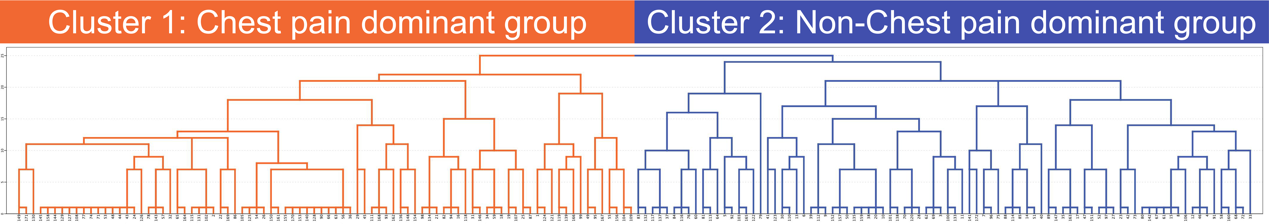


**Supplementary Fig. 1 Hierarchical cluster analysis results**

**
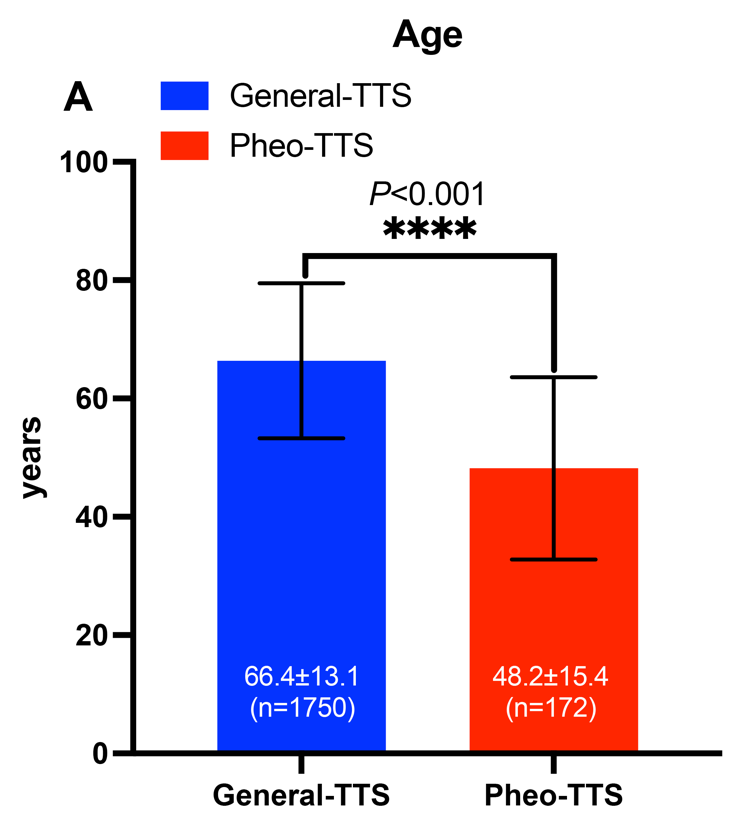

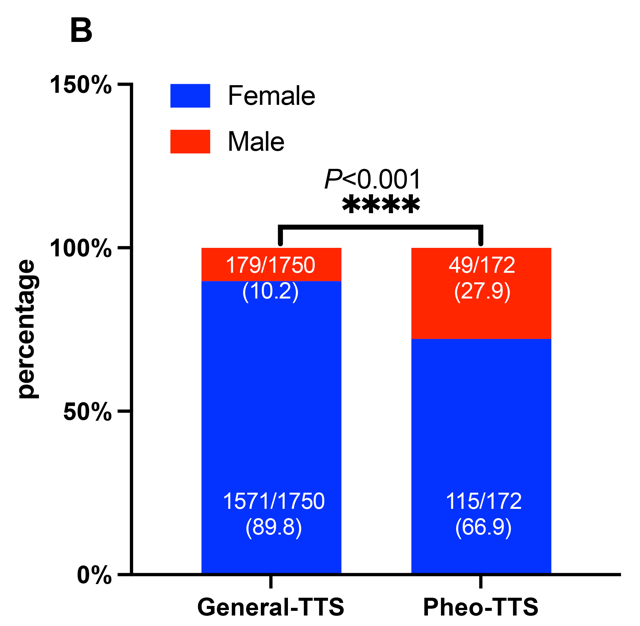

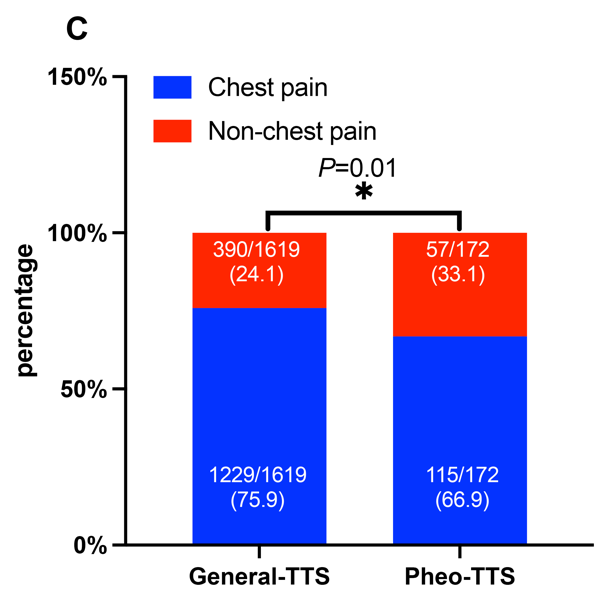

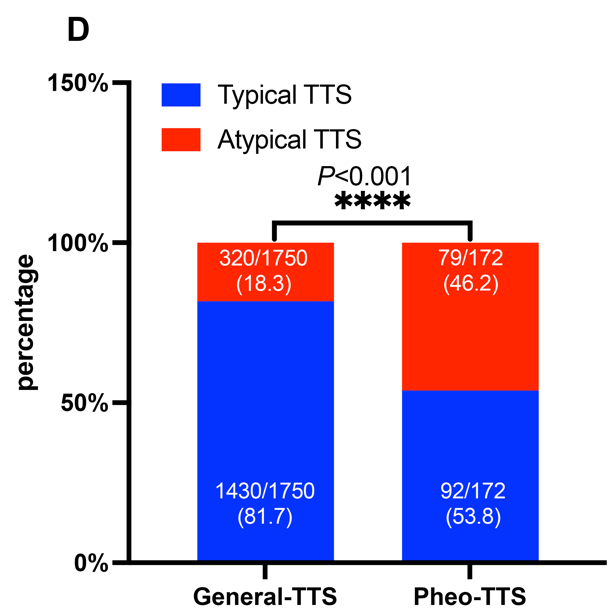

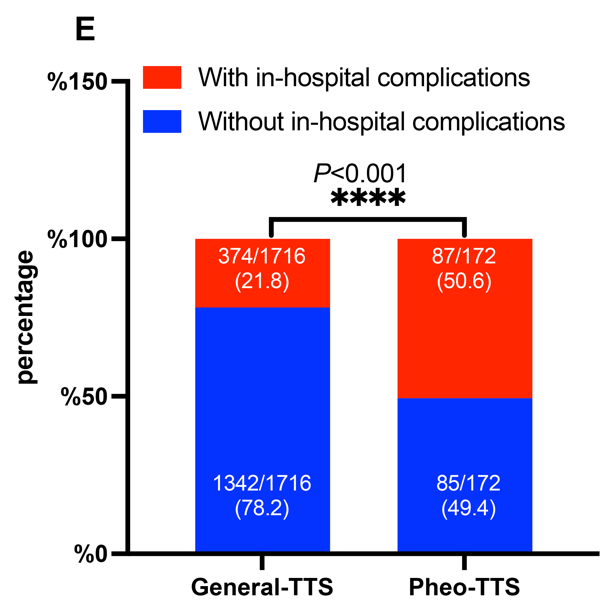
**

**Supplementary Fig. 2** **Comparison results between General-TTS and Pheo-TTS，including age(A),sex(B),incident of Chest pain(C),type of TTS(D),and occurrence of in-hospital complications(E).**
